# Author Correction: Neuronal firing rates diverge during REM and homogenize during non-REM

**DOI:** 10.1038/s41598-019-51388-2

**Published:** 2019-10-09

**Authors:** Hiroyuki Miyawaki, Brendon O. Watson, Kamran Diba

**Affiliations:** 1 Department of Psychology, University of Wisconsin-Milwaukee, P.O. Box 413, Milwaukee, WI 53211 USA; 2 Department of Physiology, Graduate School of Medicine, Osaka City University, Asahimachi 1-4-3, Abeno-ku, Osaka, 545–8585 Japan; 3 Department of Psychiatry, University of Michigan Medical School, 109 Zina Pitcher Pl, Ann Arbor, MI 48109 USA; 4 Department of Anesthesiology, University of Michigan Medical School, 1500 E Medical Center Drive, Ann Arbor, MI 48109 USA

Correction to: *Scientific Reports* 10.1038/s41598-018-36710-8, published online 24 January 2019

The Acknowledgements section in this Article was omitted. The Acknowledgements section should read:

“This project was supported by grants from the U.S. National Institutes of Health (NIMH: R01MH109170, R01MH117964 to KD, and K08MH107662 to BOW; NINDS: R21NS088798 to KD) and from JSPS KAKENHI Grant Number 17K14937 and the Kato Memorial Bioscience Foundation support to HM.”

