# Author Correction: Neuronal firing rates diverge during REM and homogenize during non-REM

**DOI:** 10.1038/s41598-021-02856-1

**Published:** 2021-12-06

**Authors:** Hiroyuki Miyawaki, Brendon O. Watson, Kamran Diba

**Affiliations:** 1 Department of Psychology, University of Wisconsin-Milwaukee, P.O. Box 413, Milwaukee, WI 53211 USA; 2 Department of Physiology, Graduate School of Medicine, Osaka City University, Asahimachi 1-4-3, Abeno-ku, Osaka, 545–8585 Japan; 3 Department of Psychiatry, University of Michigan Medical School, 109 Zina Pitcher Pl, Ann Arbor, MI 48109 USA; 4 Department of Anesthesiology, University of Michigan Medical School, 1500 E Medical Center Drive, Ann Arbor, MI 48109 USA

Correction to: Scientific Reports https://doi.org/10.1038/s41598-018-36710-8, published online 24 January 2019

The original version of this Article contained errors. The X-axis labels of the histograms “Firing rate (Hz)” were incorrectly labelled as “10^–1^” in Figure 3 B, Figure 4 A–D, Figure 5 A–C and Figure 6 C–F. In addition, in the Supplementary Information file published with this Article the X-axis labels of the histograms “Firing rate (Hz)” were incorrectly labelled as “10^–1^” in the Supplementary Figures S3 A–F, S4 A–D and S5 C–F. The correct X-axis labels now read: “10^0^”.

**Figure 3:**
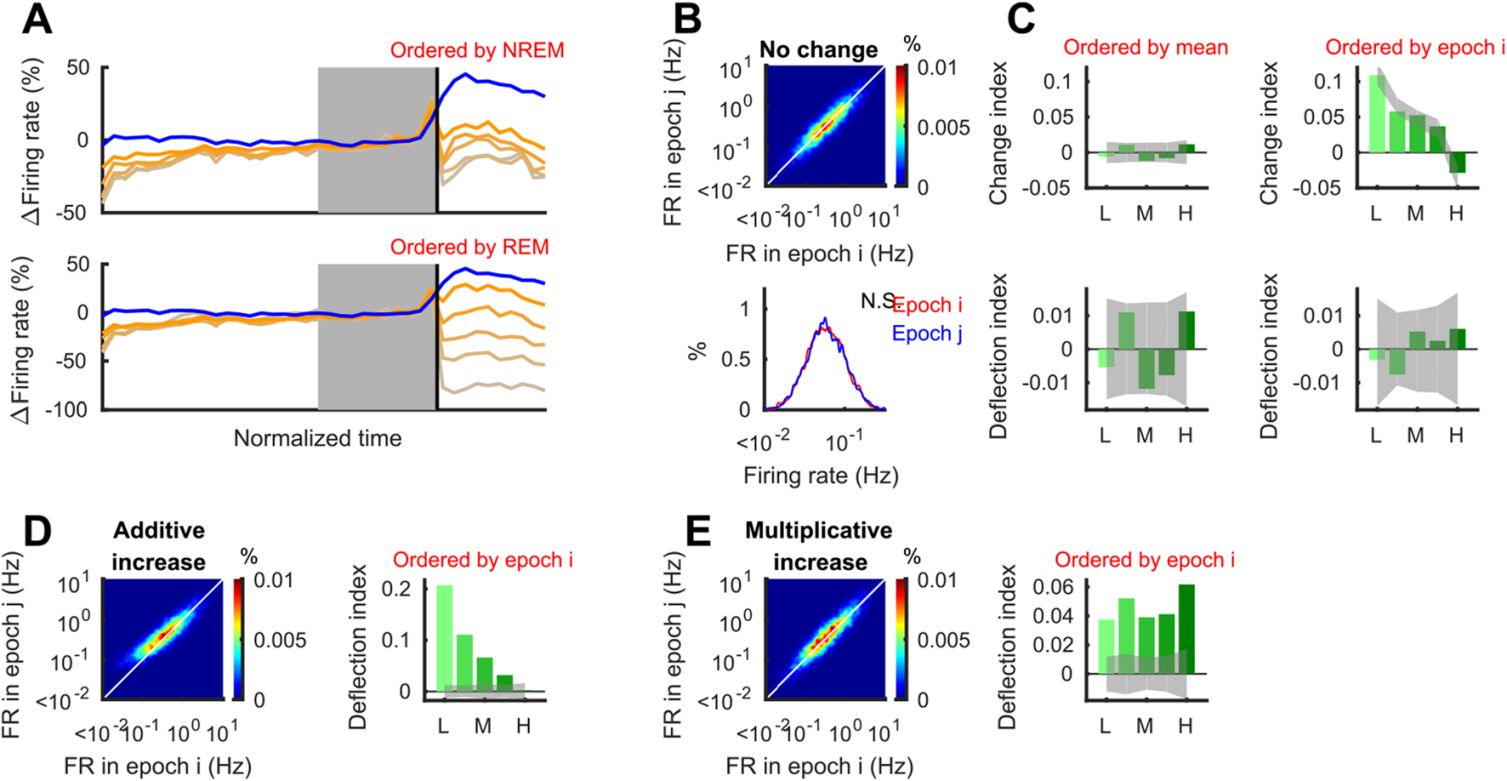
Deflection index can evaluate firing rate changes with correction for RTM. (**A**) Mean firing rates of hippocampal neurons in NREM-REM sequences as shown in Fig. 1B but different quintile separation (based on mean within NREM and REM for top and bottom, respectively). (**B**) Randomly generated firing rates with no change. (**C**) When cells are ordered based on the mean across combined states 1 and 2 (left column), there are no systematic difference on change index across quintiles. On the other hand, when cells are ordered based on state 1 alone (left column), systematic changes appear due to RTM. Deflection index (*DI*; for details see Material and Methods) can compensate for the effect of RTM (bottom panels). (**D**,**E**) In cases with non-zero changes in firing rate, *DI* is also significantly different from zero. Example of additive increase (**D**) and multiplicative increase (**E**). Gray bands indicate 95% confidence intervals obtained from shuffling (2000 times). Each example has 5000 cells whose firing rates are distributed log-normally.

**Figure 4:**
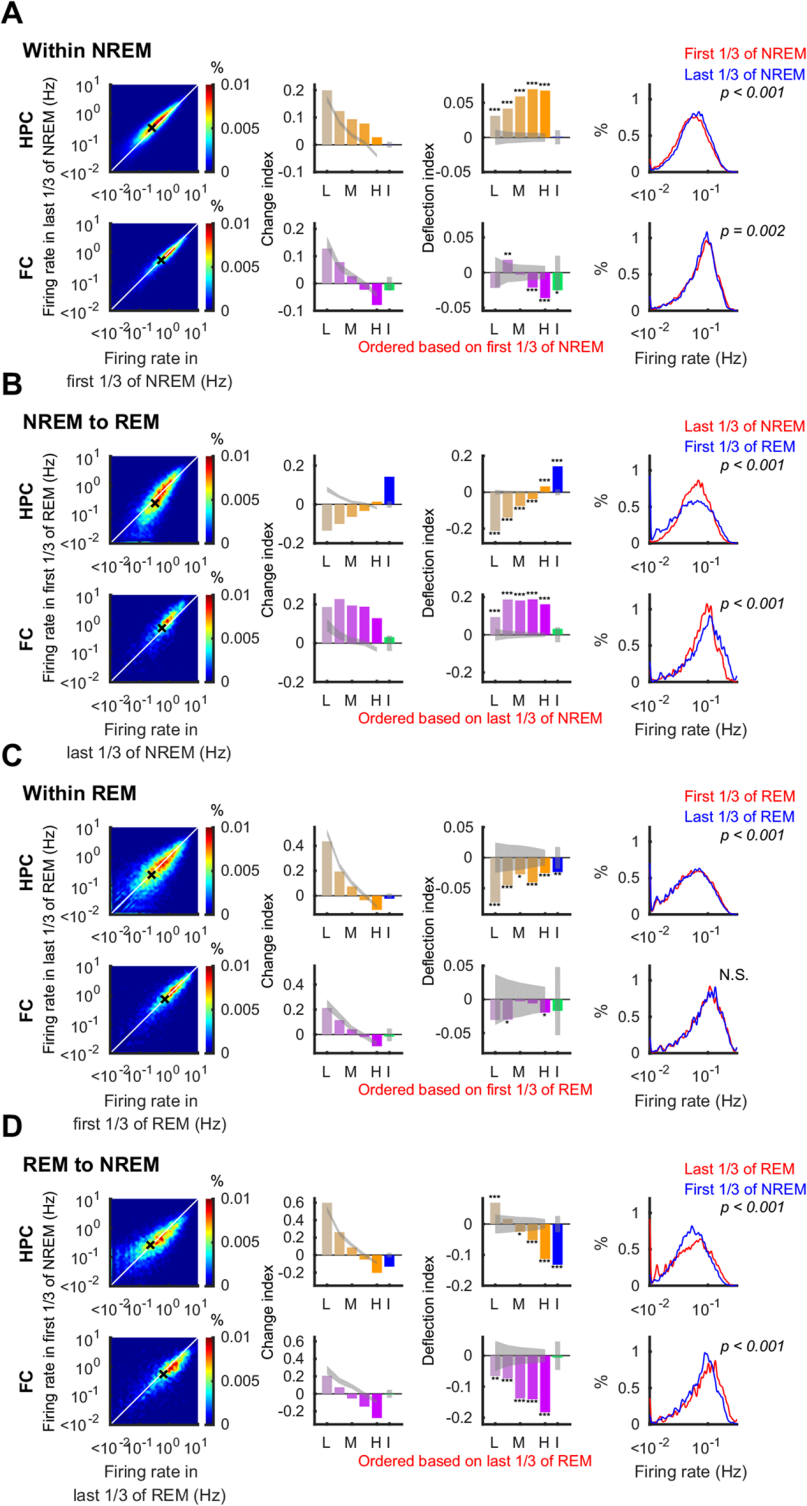
Firing rates diversify on transitions to REM and homogenize on transition to NREM. Firing rate changes within NREM (**A**), on transitions from NREM to REM (**B**), within REM (**C**), and on transitions from REM to NREM (**D**) in the hippocampus (HPC; top rows—orange) and the frontal cortex (FC; bottom rows—purple). Left panels show density (heat map) plots of firing rates. White lines indicate identity, and black crosses show means. Second and third panels illustrate change index (*CI*) and deflection index (*DI*) of each quintile of principal neurons (L: lowest quintile, M: middle quintile, H: highest quintile, yellow and purple bars) and interneurons (I, blue and green bars,) with 95% confidence interval (gray bands). Right panels show the firing rate distribution over all recorded principal neurons for the periods indicated in red and blue. P-values for the Kolmogorov–Smirnov test are indicated. *p < 0.05, **p < 0.01, ***p < 0.001.

**Figure 5:**
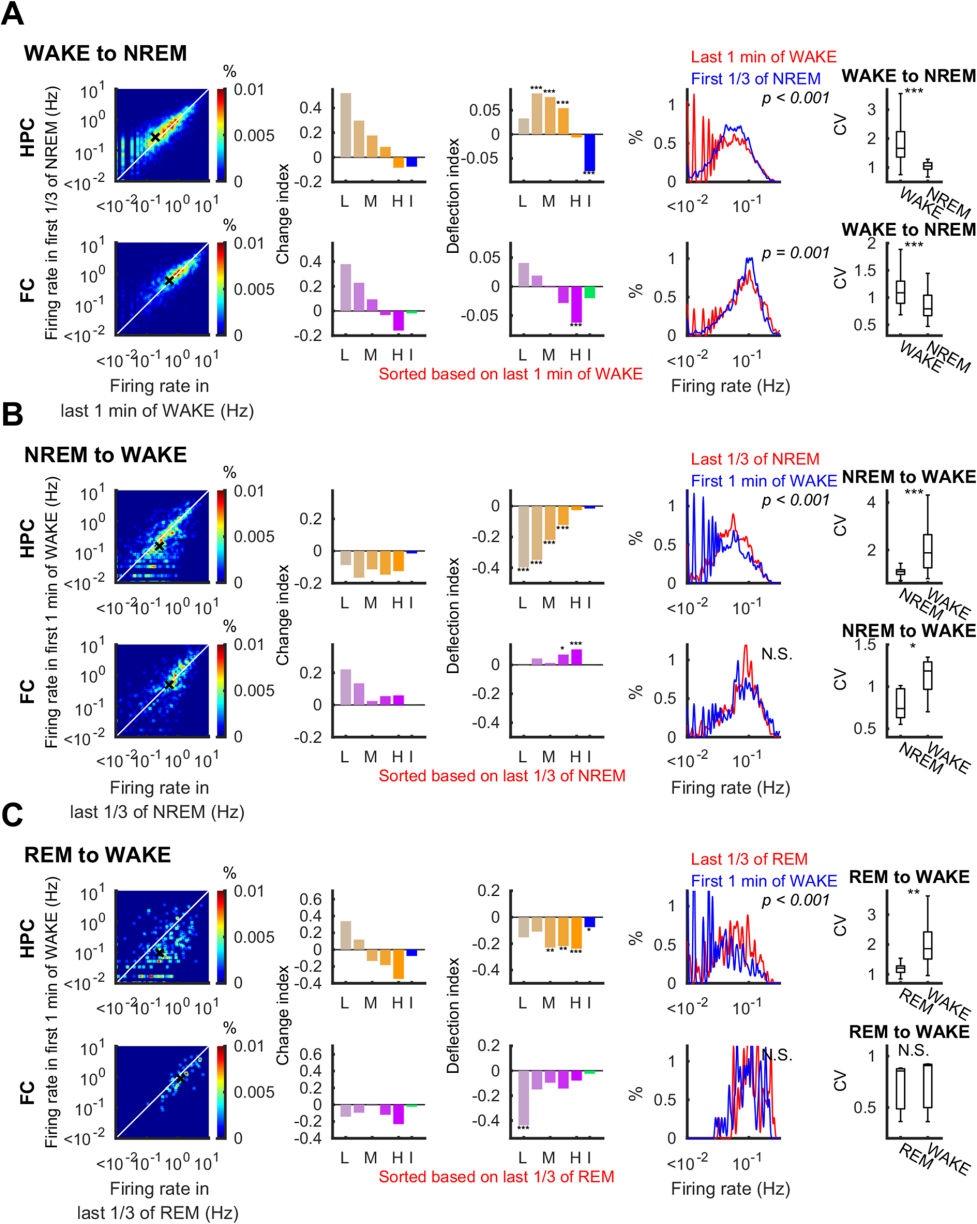
Firing rate changes at transitions between wake and sleep. Similar to Fig. 4, firing rates (left panels), change (*CI*) and deflection (*DI*) indices (second and third panels) with 95% confidence intervals of shuffle mean (sheds on the bars), firing rate distribution (fourth panels) and coefficient of variation of firing rates (right panels) on transition from WAKE to NREM (**A**), NREM to WAKE (**B**), and REM to WAKE (**C**). Top and bottom rows in each panel present data from the hippocampus (HPC) and the frontal cortex (FC), respectively. L: lowest quintile, M: middle quintile, H: highest quintile, I: interneurons. P-values for Kolmogorov–Smirnov tests are indicated on the panels in the fourth column, *p < 0.05, **p < 0.01, ***p < 0.001.

**Figure 6:**
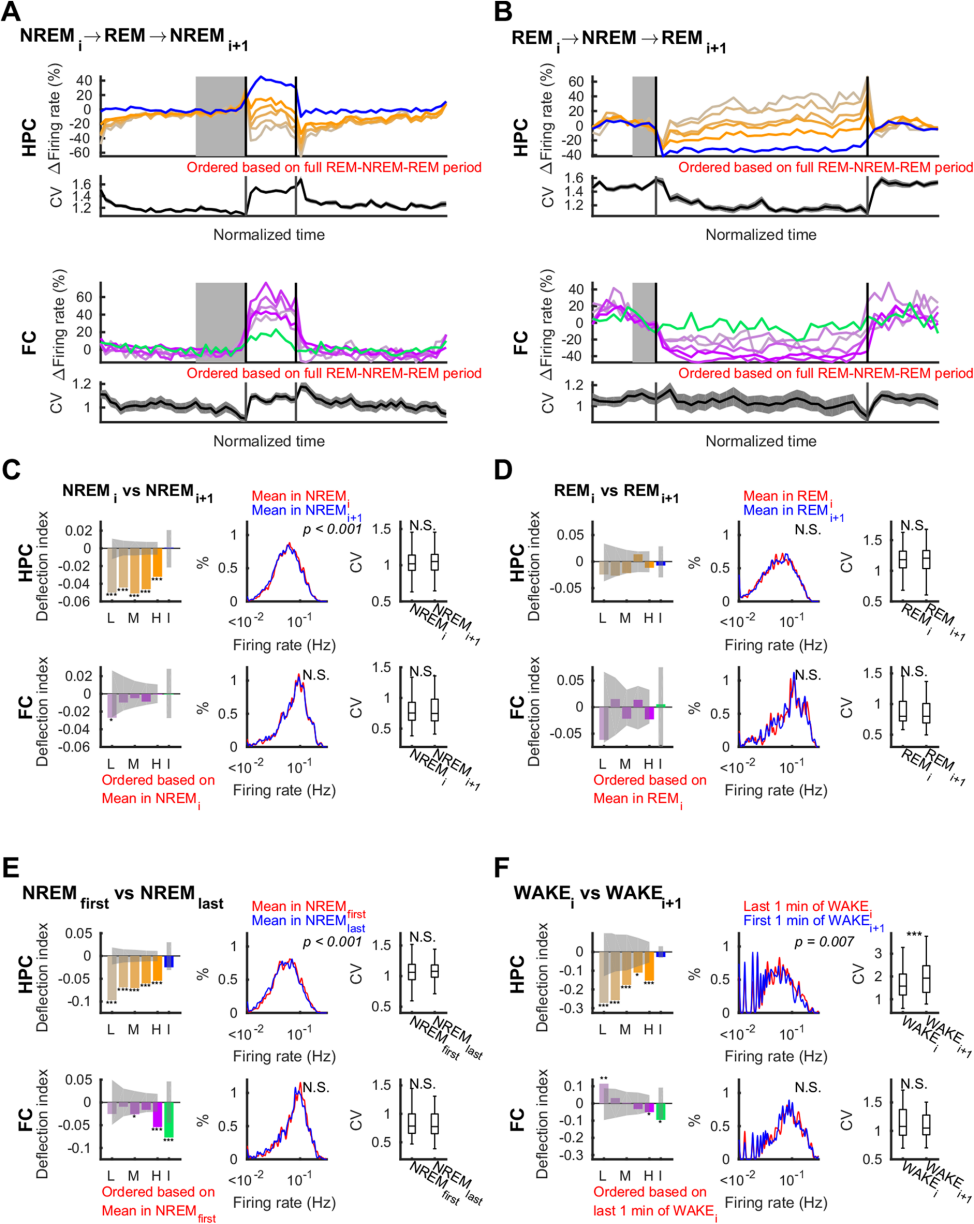
Net effects of sleep on neuronal firing distributions–analysis across state triplets. Effect of states as measured by net change from before to after that state. (**A**,**B**) Firing rates and coefficient of variation (CV) in the hippocampus (HPC; top panels) and in the frontal cortex (FC; bottom panels) in NREM_i_-REM-NREM_i+1_ triplets (**A**) and REM_i_-NREM-REM_i+1_ triplets (**B**) over time normalized for each epoch. Changes in firing rate of each quintile of pyramidal cells (orange shades) and interneurons (blue) in the hippocampus and frontal cortical principal neurons (purple shades) and interneurons (green) are relative to the mean of last third (shown in gray on the top panels and in blue on the bottom panels) of NREM_i_ in (**A**) and last third of REM_i_ in (**B**). (**C**–**F**) Deflection indices (*DI*), firing rate distributions, and CV of firing rates in (**C**) NREMs in NREM-REM-NREM triplets (**D**), REMs in REM-NREM-REM triplets, (**E**) between the first and last NREMs in each sleep, and (**F**) wake periods (last 1-min of WAKE_i_ versus first 1 min of WAKE_i+1_) separated by sleep in the hippocampus (top rows) and in the frontal cortex (bottom rows). L: lowest quintile, M: middle quintile, H: highest quintile, I: interneurons. P-values of Kolmogorov–Smirnov tests are indicated on the middle panels. Changes in CV were tested with the Wilcoxon rank sum test. Error bars and line sheds indicate SEM, sheds on bars indicate 95% confidence intervals of shuffle mean, *p < 0.05, **p < 0.01, ***p < 0.001, N.S., not significant.

Furthermore, Figures S3 B and S4 D contained errors, where the density plots did not show cross marks to indicate their mean values.

The original Figures 3, 4, 5 and 6, their accompanying legends, and the original Supplementary Information file are provided below.

Finally, in the Methods’ sub-section “Simulations”, the values for “mean” and “std” were incorrect due to a conversion error.

“To mimic the variability of real data, first we set a baseline firing rate for each cell based on a log-normal distribution obtained from hippocampal pyramidal cells during NREM (mean = 5.2 Hz, std = 7.4 Hz) and then added random (“multiplicative”) noise proportional to each cell’s firing rate in each epoch (std = 0.35 Hz).”

now reads:

“To mimic the variability of real data, first we set a baseline firing rate for each cell based on a log-normal distribution obtained from hippocampal pyramidal cells during NREM (mean = 0.59 Hz, std = 0.84 Hz) and then added random (“multiplicative”) noise proportional to each cell’s firing rate in each epoch (std = 0.35 Hz).”

These errors have now been corrected in the original Article and in the Supplementary Information file that accompanies the original Article.

## Supplementary Information

Supplementary Information.

