# Supplementary material for: Neuronal firing rates diverge during REM and homogenize during non-REM
Source: Sci Rep. 2019 Jan 24;9:689. doi: 10.1038/s41598-018-36710-8 (PMC6345798; doi:10.1038/s41598-018-36710-8)
Supplement: Supplementary file 1 — Supplementary Information [file 41598_2018_36710_MOESM1_ESM.pdf]

Supplementary Information

Neuronal firing rates diverge during REM and homogenize during non-REM.

Hiroyuki Miyawaki, PhD[1,2], Brendon O. Watson[3], Kamran Diba, PhD[1,4]*

1 Department of Psychology, University of Wisconsin-Milwaukee, P.O. Box 413, Milwaukee, WI 53211

2 Department of Physiology, Graduate School of Medicine, Osaka City University, Asahimachi 1-4-3, Abeno-ku, Osaka, 545-8585, Japan

3 Department of Psychiatry, University of Michigan Medical School, 109 Zina Pitcher Pl, Ann Arbor, MI 48109

4 Department of Anesthesiology, University of Michigan Medical School, 1500 E Medical Center Drive, Ann Arbor, MI 48109

* Corresponding: kdiba@umich.edu; Tel: 734-764-5502; Fax: 734 764-9332. Department of Anesthesiology, University of Michigan Medical School, 1500 E Medical Center Drive, Ann Arbor, MI 48109

Supplementary Figures & Legends

Figure S1

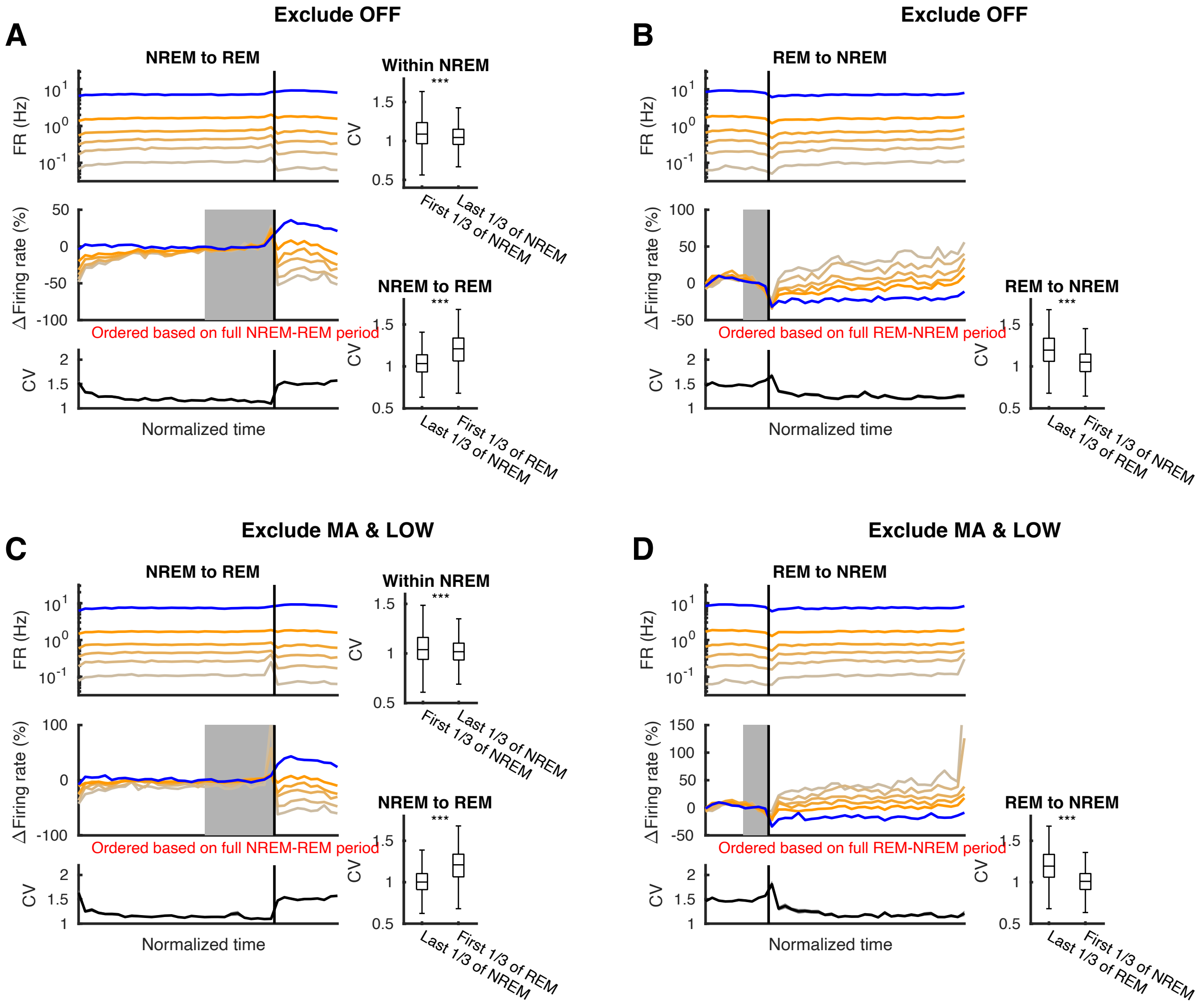

Firing rates of hippocampal neurons over sleep state transitions after exclusion of OFF or MA/LOW states. Analyses shown in Figure 1 were repeated after exclusion of OFF (A, B) or MA and LOW (C, D). Hippocampal firing rates during NREM were comparatively higher after exclusion of OFF states. Significance of CV changes were based on the Wilcoxon rank sum test. Whisker bars and line shades indicate SEM. *** $p<0.001$, N.S., not significant.

Figure S2

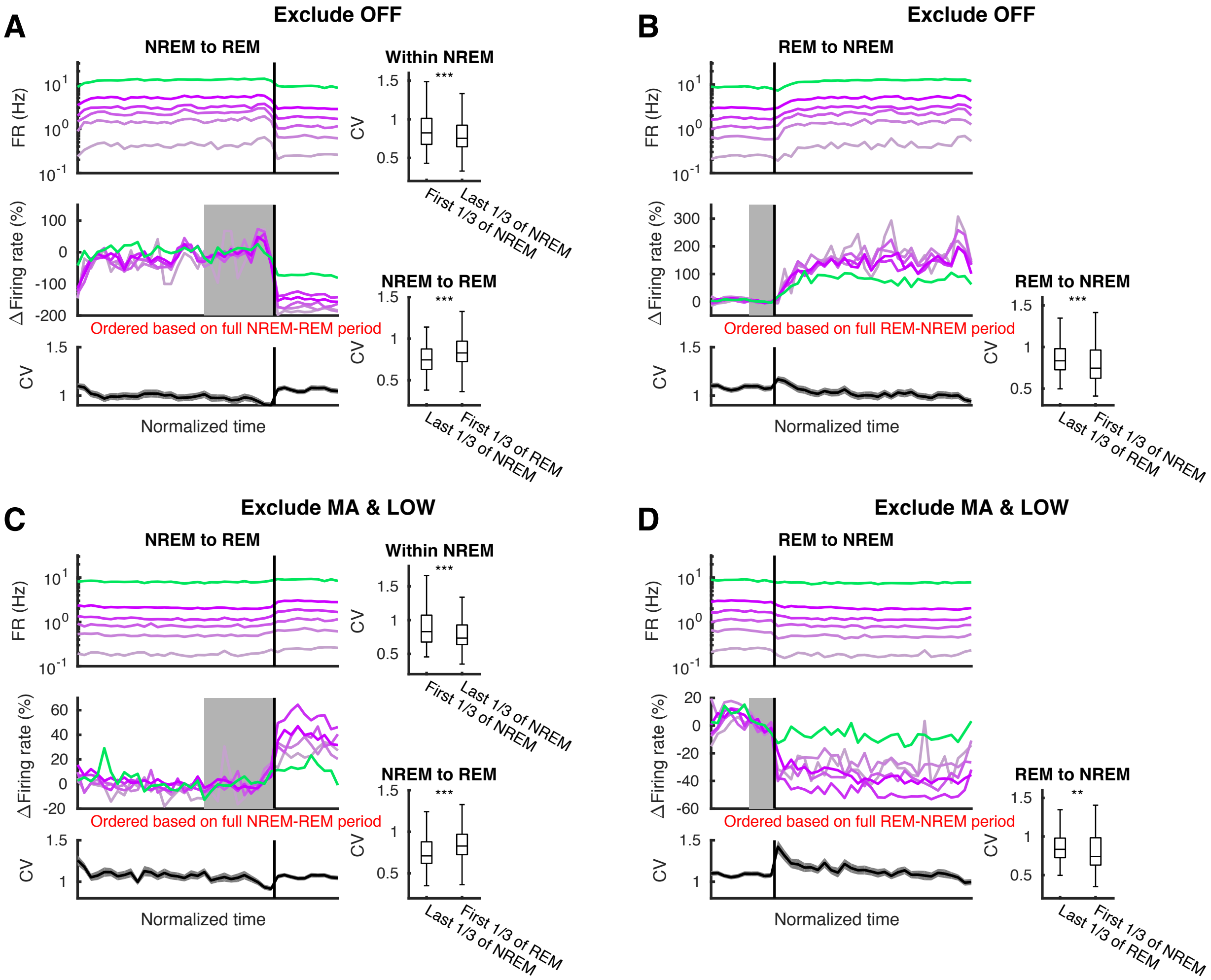

Firing rates of frontal cortical neurons over sleep state transitions after exclusion of OFF or MA/LOW states. Analyses shows in Figure 2 were repeated after exclusion of OFF (A, B) or MA and LOW (C, D). Neocortical firing rates during NREM were comparatively higher after exclusion of OFF states. Significance of CV changes were based on the Wilcoxon rank sum test. Whisker bars and line shades indicate SEM. ** $p<0.01$, *** $p<0.001$, N.S., not significant.

Figure S3

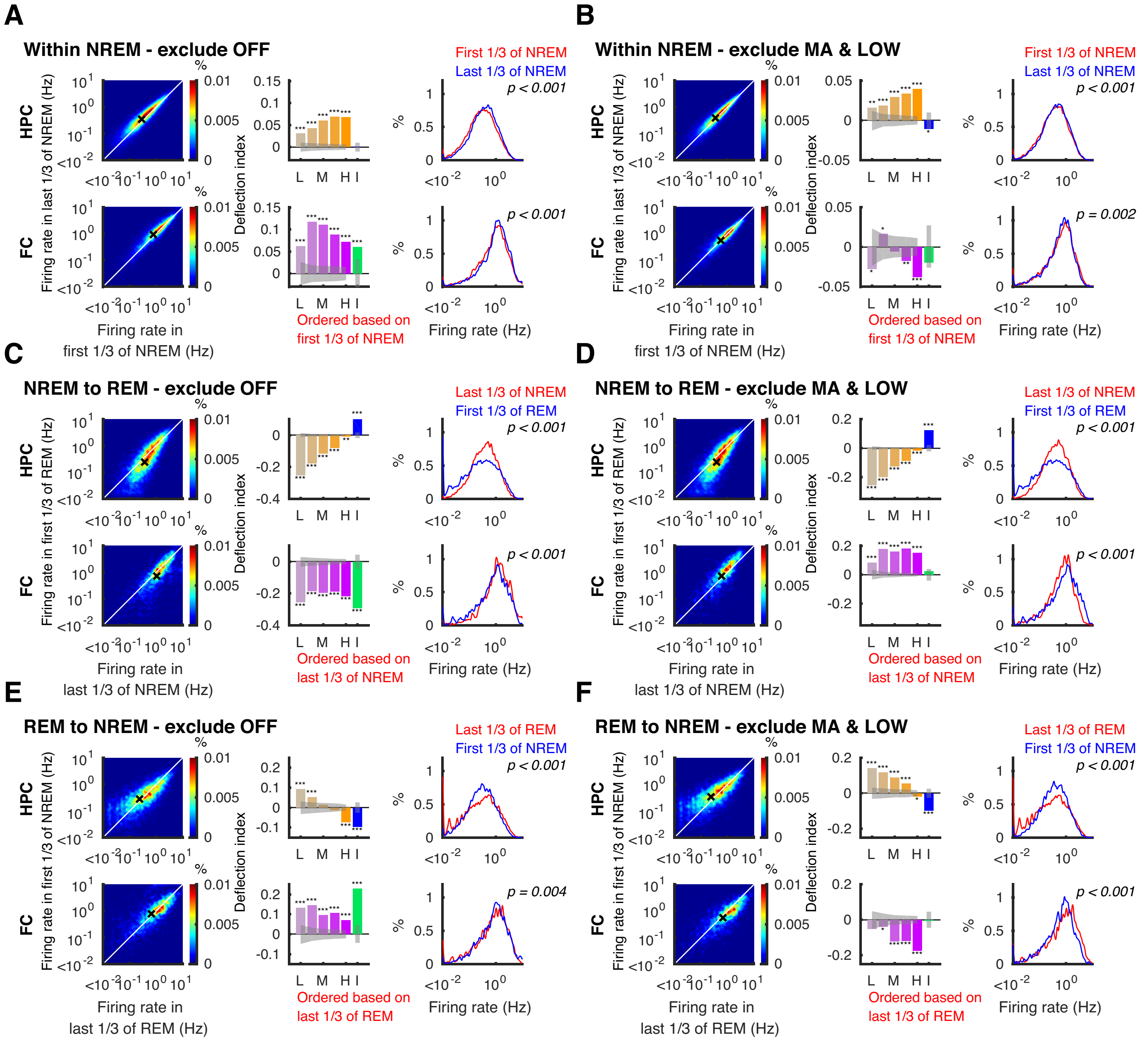

Firing rates outside of OFF or MA/LOW diversify on transitions to REM and homogenize on transition to NREM. Analyses shown in Figure 4 were repeated after exclusion of OFF (A, C, E) or MA and LOW (B, D, F). L: lowest quintile, M: middle quintile, H: highest quintile, I: interneurons. P-values from Kolmogorov-Smirnov tests are indicated on the right panels. Shaded regions on bars indicate 95% confidence intervals of shuffle mean, * p<0.05, ** p<0.01, *** p<0.001, N.S., not significant.

Figure S4

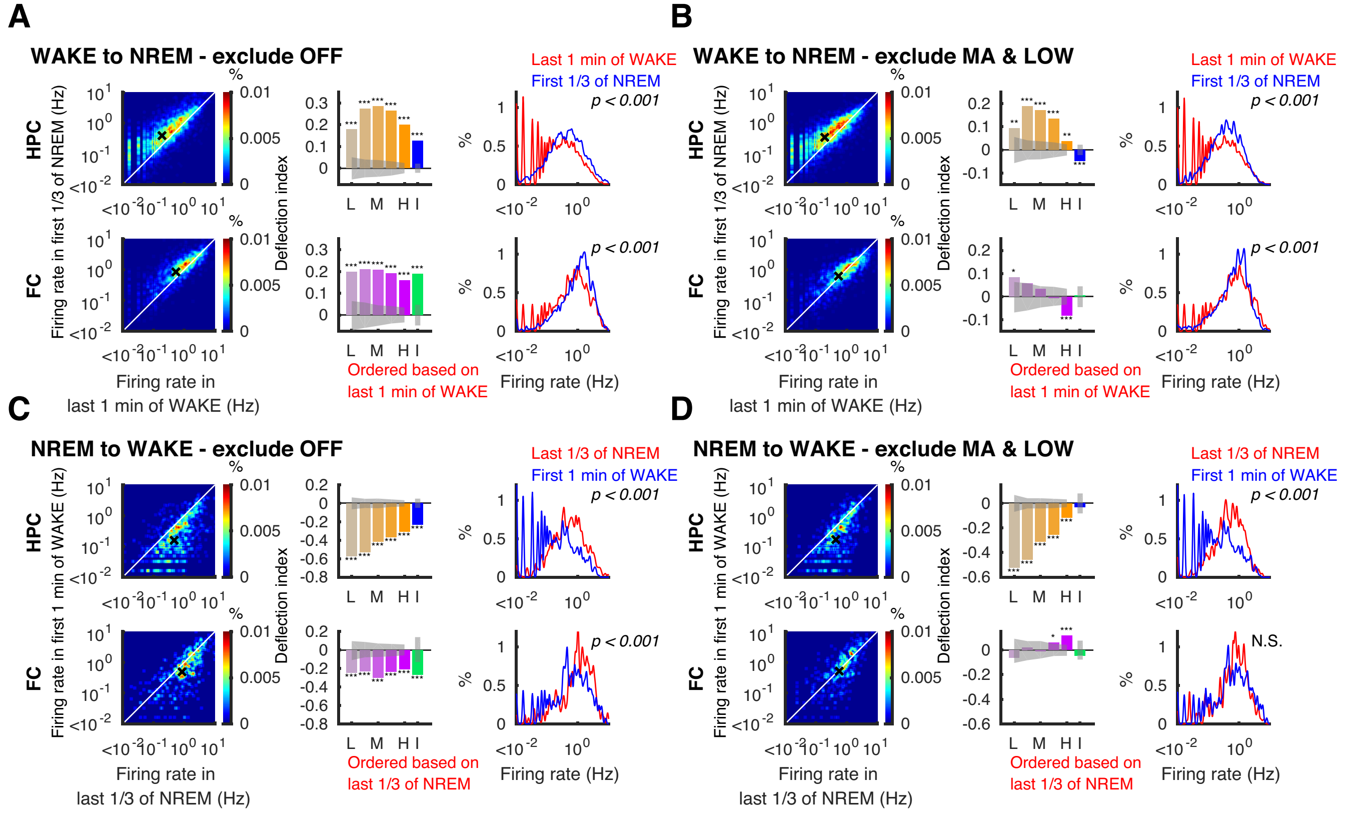

Changes in Firing rate outside of OFF or MA/LOW at transitions between wake and sleep. Analyses shown in Figure 5 were repeated after exclusion of OFF (A, C) or MA and LOW (B, D). L: lowest quintile, M: middle quintile, H: highest quintile, I: interneurons. P-values of Kolmogorov-Smirnov tests are indicated on the right panels. Shaded regions on bars indicate 95% confidence intervals of shuffle mean, * p<0.05, *** p<0.001, N.S., not significant.

Figure S5

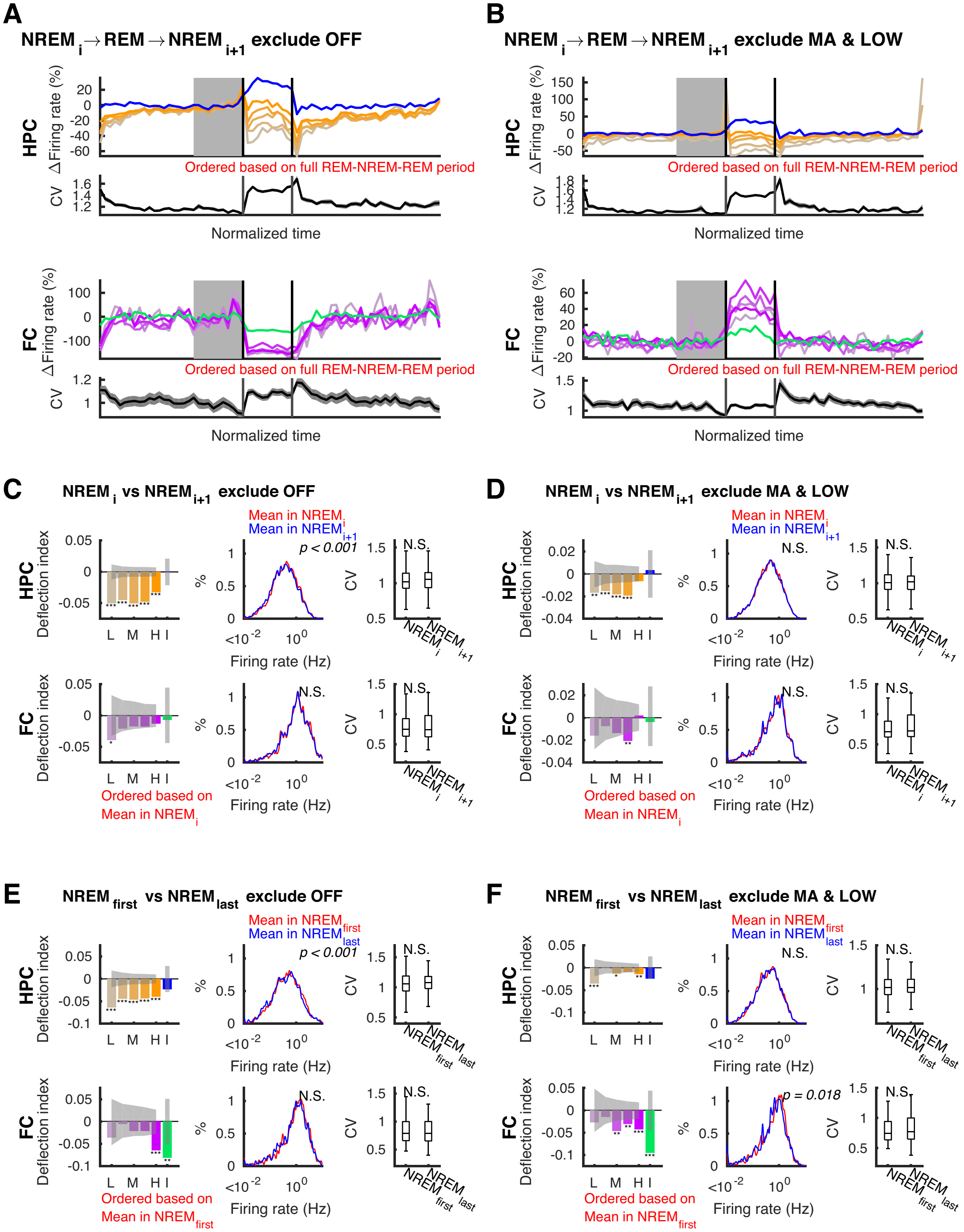

Net effects of sleep on neuronal firing distributions outside of OFF or MA/LOW. Analyses shown in Figure 6 were repeated after exclusion of OFF (A, C, E) or MA and LOW states (B, D, F). L: lowest quintile, M: middle quintile, H: highest quintile, I: interneurons. P-values from Kolmogorov-Smirnov tests are indicated on the right panels. Whiskers on box plots indicate SEM. Shaded regions on bars indicate 95% confidence intervals of shuffle mean. Changes in CV were tested with the Wilcoxon rank sum test. * p<0.05, *** p<0.001, N.S., not significant.
